# Proteomic Analysis of Colorectal Cancer: Prefractionation Strategies Using two-Dimensional Free-Flow Electrophoresis

**DOI:** 10.1002/cfg.477

**Published:** 2005-06

**Authors:** Robert L. Moritz, Anita R. Skandarajah, Hong Ji, Richard J. Simpson

**Affiliations:** Joint Proteomics Laboratory Ludwig Institute for Cancer Research (Melbourne Branch) The Walter and Eliza Hall Institute of Medical Research PO Box 2008, Royal Melbourne Hospital Parkville Victoria 3050 Australia

## Abstract

This review deals with the application of a new prefractionation tool, free-flow electrophoresis (FFE), for proteomic analysis of colorectal cancer (CRC). CRC is a leading cause of cancer death in the Western world. Early detection is the single most important factor influencing outcome of CRC patients. If identified while the disease is still localized, CRC is treatable. To improve outcomes for CRC patients there is a pressing need to identify biomarkers for early detection (diagnostic markers), prognosis (prognostic indicators), tumour responses (predictive markers) and disease recurrence (monitoring markers). Despite recent advances in the use of genomic analysis for risk assessment, in the area of biomarker identification genomic methods alone have yet to produce reliable candidate markers for CRC. For this reason, attention is being directed towards proteomics as a complementary analytical tool for biomarker identification. Here we describe a proteomics separation tool, which uses a combination of continuous FFE, a liquid-based isoelectric focusing technique, in the first dimension, followed by rapid reversed-phase HPLC (1–6 min/analysis) in the second dimension. We have optimized imaging software to present the FFE/RP-HPLC data in a virtual 2D gel-like format. The advantage of this liquid based fractionation system over traditional gel-based fractionation systems is the ability to fractionate large quantity protein samples. Unlike 2D gels, the method is applicable to both high-M_r_ proteins and small peptides, which are difficult to separate, and in the case of peptides, are not retained in standard 2D gels.


**Comparative and Functional Genomics**
*Comp Funct Genom* 2005; **6**: 236–243.
Published online in Wiley InterScience (www.interscience.wiley.com). **DOI:** 10.1002/cfg.477

**Conference Review**

### Proteomic analysis of colorectal cancer: prefractionation strategies using two-dimensional free-flow electrophoresis

Robert L. Moritz, Anita R. Skandarajah, Hong Ji and Richard J. Simpson*
*Joint Proteomics Laboratory, Ludwig Institute for Cancer Research (Melbourne Branch), The Walter and Eliza Hall Institute of Medical Research, Parkville, 3050 Victoria, Australia*

**Correspondence to: Richard J. Simpson, Joint Proteomics Laboratory, Ludwig Institute for Cancer Research, PO Box 2008, Royal Melbourne Hospital, Parkville, Victoria 3050, Australia. E-mail: richard.simpson@ludwig.edu.au*

### Abstract

**This review deals with the application of a new prefractionation tool, free-flow electrophoresis (FFE), for proteomic analysis of colorectal cancer (CRC). CRC is a leading cause of cancer death in the Western world. Early detection is the single most important factor influencing outcome of CRC patients. If identified while the disease is still localized, CRC is treatable. To improve outcomes for CRC patients there is a pressing need to identify biomarkers for early detection (diagnostic markers), prognosis (prognostic indicators), tumour responses (predictive markers) and disease recurrence (monitoring markers). Despite recent advances in the use of genomic analysis for risk assessment, in the area of biomarker identification genomic methods alone have yet to produce reliable candidate markers for CRC. For this reason, attention is being directed towards proteomics as a complementary analytical tool for biomarker identification. Here we describe a proteomics separation tool, which uses a combination of continuous FFE, a liquid-based isoelectric focusing technique, in the first dimension, followed by rapid reversed-phase HPLC (1–6 min/analysis) in the second dimension. We have optimized imaging software to present the FFE/RP-HPLC data in a virtual 2D gel-like format. The advantage of this liquid based fractionation system over traditional gel-based fractionation systems is the ability to fractionate large quantity protein samples. Unlike 2D gels, the method is applicable to both high-$M_r$ proteins and small peptides, which are difficult to separate, and in the case of peptides, are not retained in standard 2D gels.** **Copyright © 2005 John Wiley & Sons, Ltd.**

Received: 11 March 2005
Revised: 16 March 2005
Accepted: 17 March 2005

**Keywords:** free-flow electrophoresis; isoelectric focusing; mass spectrometry; colorectal cancer; proteome analysis; biomarker; reversed-phase high-performance liquid chromatography; protein purification; multidimensional

### Epidemiology of colorectal cancer (CRC)

CRC is one of the most common cancers diagnosed each year in Western countries, accounting for 13–14% of all cancer presentations [1,2] and for 10–13% of all cancer deaths [1,2]. The major impetus for research into markers of CRC is that, if detected early, when tumours are still localized [3], the projected 5 year survival rate is ∼90%. Unfortunately, most colorectal cancers (∼60%) present at an intermediate stage with a concomitant decrease in survival rates [2]. The majority of colorectal cancers (65–85%) are sporadic in nature, the result of cumulative somatic mutations [4]. Up to 30% of patients have a family history of bowel cancer, of which 6% belong to clearly defined familial genetic syndromes, such as hereditary non-polyposis colon cancer (HNPCC) and familial adenomatous polyposis (FAP). Of the sporadic risk factors, age is the most important, with the incidence increasing exponentially after age 50 [5].

Colorectal carcinomas arise from adenomas (also referred to as polyps), hence the 'adenoma–carcinoma sequence', with a lag time of ∼10 years

Copyright © 2005 John Wiley & Sons, Ltd.

[4,6,7], and the removal of these polyps has been shown to prevent colorectal cancer [7].

### Current detection methods for CRC

In the USA, UK and Australia, the general recommended screening regimen involves annual to biennial faecal occult blood tests (FOBT) and 5-yearly sigmoidoscopies [8,9]. However, effective population screening by these means is precluded by patient compliance, due to discomfort and lack of awareness of the epidemiology of colorectal cancer. If used alone, FOBT has been shown to be ineffective [10]. This type of screening is not diagnostic of CRC, but merely selects those patients who should proceed to colonoscopy.

The most widely used blood-based protein biomarker of CRC, carcinoembryonic antigen (CEA), exhibits poor sensitivity for screening for the early detection of the disease and is mainly employed in post-operative detection for recurrence and monitoring of metastasis [11,12]. Whilst a single marker may lack sensitivity and specificity for detection, combining markers improves these parameters [13,14].

### Proteomics tools

Current proteomics research can be defined as two contrasting but complementary strategies, cell-mapping proteomics and protein expression proteomics [15,16].

### Cell-mapping proteomics

Cell-mapping proteomics aims to define protein–protein interactions to build a picture of the complex networks that constitute intracellular signalling pathways. Many genetic mutations associated with cancer progression affect genes encoding proteins in signalling pathways, highlighting the importance of defining these signalling networks [17]. For example, Pandey *et al.* treated HeLa cells with either epidermal growth factor or platelet-derived growth factor (PDGF), and used anti-phosphotyrosine immunoprecipitation to concentrate a range of proteins that were subsequently phosphorylated [18]. The analysis revealed the role of vav-2, as well as a number of other proteins, in growth factor signalling. Alternatively, Lewis *et al.* selectively activated or inhibited the mitogen-activated protein kinase (MAPK) pathway and used a proteomic approach to identify 20 novel targets of MAPK signalling [19]. Affinity capture techniques have also been used to identify the anti-apoptotic protein DIABLO/SMAC [20] and binding proteins for suppressors of cytokine signalling, SOCS [21]. Although the scope of this review does not cover the full range of possibilities available, these few examples show the utility of cell-mapping proteomics.

### Protein-expression proteomics

Protein expression analysis monitors global expression of large numbers of proteins within a cell type or tissue and quantitatively identifies how patterns of expression change in different circumstances. Global protein profiles can be produced for normal compared with tumour cells in a given tissue, or for cells before and after treatment with a specific drug. Currently, this is the most widely used model of proteomics and is largely dependent upon two-dimensional gel electrophoresis (2DE) for visualization of protein profiles. Expression proteomics is the protein equivalent of DNA microarray analysis. Like DNA microarrays, it has the advantage of being non-prejudicial and could define unexpected ways in which known proteins regulate cellular responses. Major limitations of the 2DE system include an inability to detect proteins of medium to low abundance, as well as a limited apparent molecular mass range ($M_r$), where molecules smaller than 10 K are generally lost. This has prompted much interest in non-2DE approaches for studying global protein profiles.

### Correlation of mRNA transcripts and protein expression levels

In the search for tumour progression markers or anticancer drug targets, there has been a concerted effort to define gene expression profiles at the transcript level [22,23]. However, it is clear that mRNA expression data alone are insufficient to predict functional outcomes for the cell, as they provide very little information about activation state, post-translational modification or localization of corresponding proteins. Moreover, there are numerous

Copyright © 2005 John Wiley & Sons, Ltd. 

reports highlighting the disparity between mRNA transcript and protein expression levels [24,25]. Thus, at the very least, mRNA expression studies must be supported with proteomic information in an integrated approach to provide a complete picture of how cells are altered during malignant transformation [25,26].

### Dynamic range of protein abundances

Several technical issues need to be addressed before proteomics can realize its full potential for protein expression profiling of complex proteomes such as cells and tissues [27,28]. Foremost is the problem of dynamic range of protein abundances. For instance, the dynamic range of protein abundances in blood is thought to be $\sim 10^{10}$ [29]. This makes it extremely challenging to visualize low-abundance proteins and peptides in complex proteomes such as blood, let alone identify them using current mass spectrometry (MS)-based identification methods [30–32]. For example, the most abundant protein in human plasma is serum albumin (HSA), present at 40–50 mg/ml, whereas some of the least abundant proteins, such as cytokines [33] and protease biomarkers (e.g. the prostate-specific antigen [34]), are present at ~10 and ~3 pg/ml levels, respectively. Given that the current sensitivity of routine protein and peptide identification by MS–MS is ~500 amol, in order to obtain 500 amol of IL-6, approximately ~1.5 ml plasma would be required for the initial fractionation step. However, this amount of plasma would also contain 1.4 µM (90 mg) HSA, which is $6.2 \times 10^9$ (w/w) in excess of IL-6. This is a formidable quantity of protein to fractionate and presents a challenge to current purification schemes, where extensive pre-fractionatation/depletion strategies need to be invoked in order to reveal low-abundance proteins. However, this also assumes that the protein of interest is homogenous and a 100% recovery is obtained through all fractionation steps. For IL-6, which is extensively post-translationally modified (p$I$ range 5–7, $M_r$ range 22–29 K [33,35,36]), much larger quantities of plasma would be required to obtain a single enriched population of IL-6 molecules for identification purposes. Of equal importance is the problem that proteins exhibit tremendous heterogeneity with respect to size, charge, post-translational modifications and solubility. Consequently, a wide range of protein separation methods, or combinations thereof, i.e. multidimensional separation strategies, are usually required for the comprehensive analysis of complex proteomes.

For several decades, 2DE [37,38] has been the only proteomics technique that has permitted the separation of thousands of proteins in a single experiment [39,40]. However, the dynamic range of protein abundances that can be separated by 2DE is $\sim 10^4$, which is inadequate for cell types and tissues such as blood.

### Prefractionation approaches in proteome analysis

Several approaches for overcoming the protein dynamic range obstacle have been recently described, viz. (a) the disassembly of the macromolecular architecture of cells into their constituent organelles, macromolecular structures and multiprotein complexes allowing subproteome analysis [41,42]; (b) enrichment of cellular components using differential detergent fractionation [43,44] or molecular weight fractionation [45]; (c) depletion of abundant proteins using non-biospecific chromatography [46], immunoaffinity chromatography using specific antibodies against abundant proteins [47], and removal of IgG from serum using immobilized protein-A or — G columns [48–52]; (d) selective precipitation, e.g. with trifluoracetic acid/acetone [53] prior to 2DE; and (e) various chromatographic [54,55] and electrophoretic methods [56].

Prefractionation of proteins from large volumes, as a prelude to subsequent analysis using 2DE, can be performed by preparative polyacrylamide gel electrophoresis (PAGE) or size-exclusion chromatography on the basis of $M_r$ [57,58]. Alternative electrophoretic prefractionation methods based upon solution-phase IEF, rather than gel-based IEF [59] used in 2DE, have been developed. These non-gel-based IEF methods can accommodate large sample volumes and amounts in contrast to gel-based IEF methods. Preparative IEF as a prefractionation technique was first proposed by Bier's laboratory [60,61]. To overcome problems associated with the original device, Righetti and colleagues [62] developed a multicompartment electrolyser in which each compartment was separated

Copyright © 2005 John Wiley & Sons, Ltd. 

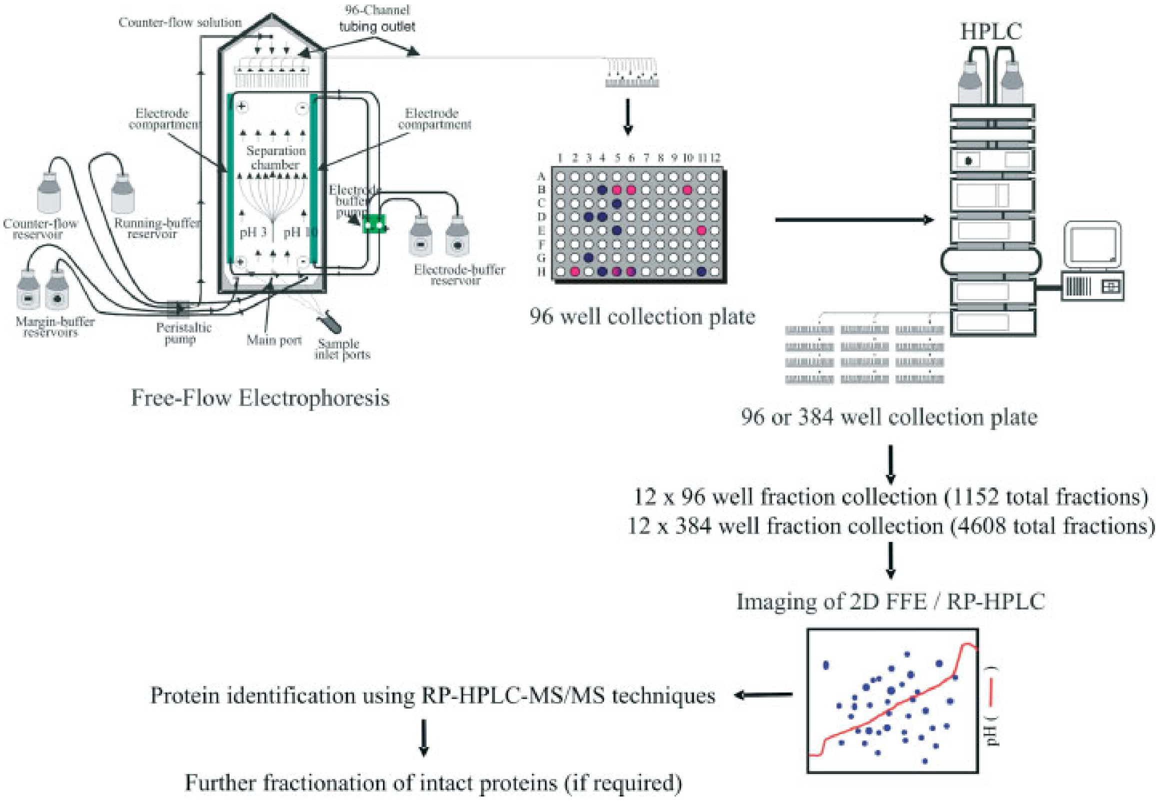

**Figure 1.** Schematic representation of the continuous FFE apparatus coupled off-line to RP-HPLC. Dimensions of focusing chamber: 50 × 10 × 0.04 cm (10 cm between electrodes, 45 cm electrode length). For analytical imaging separations, a portion of each first-dimension FFE-IEF fraction (50 µl/total volume ∼2 ml) was injected directly from the 96 deep-well plate using the Agilent 1100 HPLC equipped with a well-plate autosampler and samples collected automatically into a multi-plate fraction collection system by a polyacrylamide gel membrane, each with a defined pH. A microscale liquid-phase IEF prefractionation method that is also based upon a multicompartment apparatus (4.7 ml total volume, 650 µl/chamber) has recently been described by Speicher *et al.* [63]. In this apparatus the chambers are separated by thin polyacrylamide gels containing ampholyte mixtures at specific pH values. For a recent review of prefractionation techniques in proteome analysis, see Righetti *et al.* [64] and Simpson [28].

### Free-flow electophoresis (FFE)

FFE, first described by Hannig, [65,66], is a separation device that continuously streams a sample into a carrier ampholine solution flowing as a thin laminar film (0.3–1.0 mm) between two flat plates (see Figure 1). By introducing an electric field perpendicular to the direction of flow, cellular organelles, proteins and low $M_r$ species such as peptides can be separated by IEF according to their different p*I* values and subsequently collected for further analysis [67,68]. Previously, we reported an uncoupled FFE-IEF/SDS-PAGE strategy for separating cytosolic proteins from a human colon carcinoma cell line for subsequent identification by on-line RP-HPLC/electrospray-ionization (ESI)-ion trap MS [69]. We have further refined this strategy to provide a complete liquid-based fractionation strategy by introducing off-line rapid RP-HPLC (1–6 min separation times) as a second dimension for each of the FFE fractions. An example of a complex protein separation, such as a cell lysate, using this method is shown in Figure 2. Unlike 2DE, this technique is suitable for separating low-$M_r$ proteins and polypeptides and does not have the problem of sample loadability, due to the

Copyright © 2005 John Wiley & Sons, Ltd. 

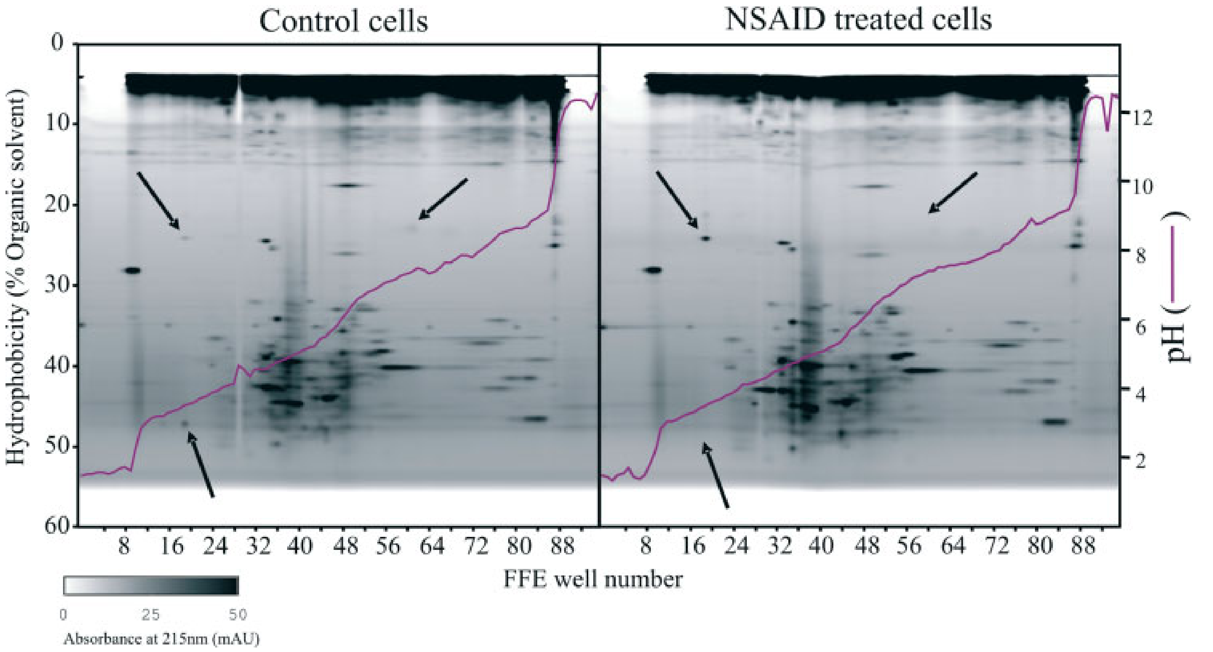

**Figure 2.** Two-dimensional FFE-IEF/RP-HPLC separation of cytosolic proteins from untreated and non-steriod anti-inflammatory drug (NSAID)-treated human LIM 1215 colorectal cell line. (A) Sample: 1 mg untreated LIM 1215 cytosolic proteins dissolved in 1 ml IEF running buffer containing 0.2% (w/v) HPMC, 0.4% (v/v) Servalyte pH 3–10 loaded at 1.4 ml/h at 1500 V, 15 mA and collected into a deep 96-well plate. Second dimension RP-HPLC separation of FFE-IEF fraction. (B) Sample: 1 mg NSAID-treated LIM1215 cytosolic proteins. FFE-IEF and second dimension RP-HPLC separation are as in (A); proteins indicated by arrows were dysregulated by NSAID treatment of LIM 1215 cells (R.L. Moritz, manuscript in preparation)

ease by which the method can accommodate large sample volumes [70]. FFE can be performed over both broad and narrow ranges of pH by the judicious choice of ampholytes in the first dimension step. Additionally, we have developed software to present the chromatographic output as a single 2D plot (virtual 2D analysis) for quick visual evaluation and 'spot' matching of fractionated proteins.

The ability to fractionate low-$M_r$ compounds is an important feature of this 2D liquid-based FFE-IEF/RP-HPLC method, because techniques designed for this purpose are under represented in the armory of current proteomic separation tools. 2D gel-based systems, multicompartment and column-based separation systems are also limited by the quantity of bulk starting material that can be loaded in the first dimension (IPG). This limitation in fractionation of starting material can hamper efforts to mine complex tissues.

The advantages of this system can be summarized as follows: (a) protein and/or peptide separations in the first dimension (IEF) are performed in a liquid phase and, unlike other multicompartment electroanalysers, are not restricted by passaging through any barrier or matrix; (b) the system is truly preparative by not being sample-limited, and separation efficiency is maintained by continual flushing of the separated sample; and (c) the FFE-IEF/RP-HPLC system is capable of separating compounds of low $M_r$ (e.g. peptides) as well as high $M_r$ (e.g. native proteins and their multimeric complexes) over a broad pH range. For those proteins (especially membrane proteins) that exhibit poor solubility at or near their p$I$ value, an appropriate buffer (e.g. amino acids as well as detergents in the case of membrane protein separations) can be incorporated in the FFE counterflow media prior to collection in the 96-well plate to minimize the time that such proteins stand at their p$I$ value [69]. The high-resolving power produced in the first dimension IEF step, where very narrow range pH gradients can easily be generated, coupled to the high resolution of modern RP-HPLC stationary phases, extends the resolving power of this 2D protein separation system over other previously described 2D systems based solely on coupled

Copyright © 2005 John Wiley & Sons, Ltd. 

HPLC columns [71,72]. In the case of high-$M_r$ proteins and very hydrophobic proteins such as membrane proteins, the RP-HPLC stationary phases can be substituted by other chromatographic modes, such as hydrophobic interaction chromatography or hydroxyapatite stationary phases to extend the power of the method to cover classes of proteins that are refractory to RP chromatography.

The advantage of the 2D liquid-based FFE/RP-HPLC system over traditional gel-based systems is that complex mixtures of low-$M_r$ compounds, such as tryptic peptides, can be fractionated, whereby peptides are fractionated into discrete pools with increasing pH values that vary one from another by $\sim$0.02 pH units. The ease and rapid determination of the apparent peptide p$I$ value can be achieved by measuring the pH of these pools using a laboratory combination pH electrode. By applying a peptide's p$I$ determinant to high mass accuracy ($\leqslant$1 ppm) peptide-mass fingerprinting, [73–75] the discrimination of peptides differing by small mass units or even isobaric peptides is feasible [76]. Peptide mass, as determined by current high mass accuracy mass spectrometers, by itself as the only parameter for peptide identification in large genome databases, is insufficient for high-confidence protein identification and is no longer considered a valid approach to protein identification by the editors of proteomics journals [77]. The use of a peptide's p$I$ value combined with MS–MS data also provides a powerful qualifier for peptide identifications, as well as decreasing the peptide search pool. The latter can be achieved by minimizing the number of peptides used to search with, by only considering those peptides that are within a discrete p$I$ range in conjunction with the mass range defined by the accuracy of the mass spectrometer used.

### Concluding remarks

The 2D liquid based FFE-IEF/RP-HPLC method described here promises to play a key role in the analysis of complex protein and low-$M_r$ compounds, the latter being largely under represented in most proteome studies to date. Interestingly, low-$M_r$ proteins and peptides are thought to contain a rich source of previously undiscovered biomarkers of disease [78]. Although current claims regarding the potential of proteomics to define cancer-related molecules might outnumber reports of concrete achievement, both global-expression [79,80] and cell-mapping proteomics [19–21] have contributed to our understanding of cell biology and disease. To date, several proteins have been identified from colon tumour samples and patient blood that can potentially be used for biomarkers for the identification of early onset colorectal cancer [81,82]. Validation of these identifications must be performed before these proteins can be ascribed as diagnostic biomarkers [83]. It is anticipated that further proteomics studies aimed at identifying specific proteins present in biological specimens of diseased patients may reveal a panel of proteins that correlate with aberrant growth specific or individual cancer subtypes. When used in combination, such a cohort of biomarkers may provide for a high-sensitivity and -specificity predictive assay with minimal invasion to the patient, thereby allowing for population-based screening.

### Acknowledgement

Funding was provided by the Australian National Health and Medical Research Council under Program Grant No. 280912.

### References

1. Australian Institute of Health and Welfare (AIHW) & Australasian Association of Cancer Registries (AACR) (ed). 2003. *Cancer in Australia 2000*. AIHW: Canberra; **http://www.aihw.gov.au**
2. American Cancer Society (ed). 2004. *Cancer Facts and Figures*. American Cancer Society: Atlanta; **http://www.cancer.org**
3. Etzioni R, Urban N, Ramsey S, *et al*. 2003. The case for early detection. *Nat Rev Cancer* **3**: 243–252.
4. Fearon ER, Vogelstein B. 1990. A genetic model for colorectal tumorigenesis. *Cell* **61**: 759–767.
5. Chung DC. 2000. The genetic basis of colorectal cancer: insights into critical pathways of tumorigenesis. *Gastroenterology* **119**: 854–865.
6. Fearon ER. 1994. Molecular genetic studies of the adenoma–carcinoma sequence. *Adv Intern Med* **39**: 123–147.
7. Winawer SJ, Zauber AG, Ho MN, *et al*. 1993. Prevention of colorectal cancer by colonoscopic polypectomy. The National Polyp Study Workgroup. *N Engl J Med* **329**: 1977–1981.
8. Cancer Research UK (ed). 2004. *Large Bowel Cancer Factsheet*; **http://www.cancerresearchuk.org**
9. American Cancer Society (ed.) 2004. *Cancer Facts & Figures*. American Cancer Society: Atlanta, GA.
10. Collins JF, Lieberman DA, Durbin TE, Weiss DG. 2005. Accuracy of screening for fecal occult blood on a single stool sample obtained by digital rectal examination: a comparison with recommended sampling practice. *Ann Intern Med* **142**: 81–85.

Copyright © 2005 John Wiley & Sons, Ltd. 

11. Crawford NP, Colliver DW, Galandiuk S. 2003. Tumor markers and colorectal cancer: utility in management. *J Surg Oncol* **84**: 239–248.
12. Duffy MJ, van Dalen A, Haglund C, *et al*. 2003. Clinical utility of biochemical markers in colorectal cancer: European Group on Tumour Markers (EGTM) guidelines. *Eur J Cancer* **39**: 718–727.
13. Holten-Andersen MN, Christensen IJ, Nielsen HJ, *et al*. 2002. Total levels of tissue inhibitor of metalloproteinases 1 in plasma yield high diagnostic sensitivity and specificity in patients with colon cancer. *Clin Cancer Res* **8**: 156–164.
14. Hall NR, Finan PJ, Stephenson BM, Purves DA, Cooper EH. 1994. The role of CA-242 and CEA in surveillance following curative resection for colorectal cancer. *Br J Cancer* **70**: 549–553.
15. Blackstock WP, Weir MP. 1999. Proteomics: quantitative and physical mapping of cellular proteins. *Trends Biotechnol* **17**: 121–127.
16. Simpson RJ, Dorow DS. 2001. Cancer proteomics: from signaling networks to tumor markers. *Trends Biotechnol* **19**: S40–S48.
17. Downard J. 2001. The ins and outs of signalling. *Nature* **411**: 759–762.
18. Pandey A, Podtelejnikov AV, Blagoev B, *et al*. 2000. Analysis of receptor signaling pathways by mass spectrometry: identification of vav-2 as a substrate of the epidermal and platelet-derived growth factor receptors. *Proc Natl Acad Sci USA* **97**: 179–184.
19. Lewis TS, Hunt JB, Aveline LD, *et al*. 2000. Identification of novel MAP kinase pathway signaling targets by functional proteomics and mass spectrometry. *Mol Cell* **6**: 1343–1354.
20. Verhagen AM, Ekert PG, Pakusch M, *et al*. 2000. Identification of DIABLO, a mammalian protein that promotes apoptosis by binding to and antagonizing IAP proteins. *Cell* **102**: 43–53.
21. Zhang JG, Farley A, Nicholson SE, *et al*. 1999. The conserved SOCS box motif in suppressors of cytokine signaling binds to elongins B and C and may couple bound proteins to proteasomal degradation. *Proc Natl Acad Sci USA* **96**: 2071–2076.
22. Ross DT, Scherf U, Eisen MB, *et al*. 2000. Systematic variation in gene expression patterns in human cancer cell lines. *Nat Genet* **24**: 227–235.
23. Scherf U, Ross DT, Waltham M, *et al*. 2000. A gene expression database for the molecular pharmacology of cancer. *Nat Genet* **24**: 236–244.
24. Gygi SP, Rochon Y, Franza BR, Aebersold R. 1999. Correlation between protein and mRNA abundance in yeast. *Mol Cell Biol* **19**: 1720–1730.
25. Tian Q, Stepaniants SB, Mao M, *et al*. 2004. Integrated genomic and proteomic analyses of gene expression in mammalian cells. *Mol Cell Proteom* **3**: 960–969.
26. Cox B, Kislinger T, Emili A. 2005. Integrating gene and protein expression data: pattern analysis and profile mining. *Methods* **35**: 303–314.
27. Simpson RJ. 2003. *Proteins and Proteomics: A Laboratory Manual*. Cold Spring Harbor Laboratory Press: Cold Spring Harbor, NY.
28. Simpson RJ. 2004. *Purifying Proteins for Proteomics: A Laboratory Manual*. Cold Spring Harbor Laboratory Press: Cold Spring Harbor, NY.
29. Anderson NL, Polanski M, Pieper R, *et al*. 2004. The human plasma proteome: a non-redundant list developed by combination of four separate sources. *Mol Cell Proteomics*. **3**: 311–326.
30. Domon B, Alving K, He T, Ryan TE, Patterson SD. 2002. Enabling parallel protein analysis through mass spectrometry. *Curr Opin Mol Ther* **4**: 577–586.
31. Patterson SD. 2003. Proteomics: evolution of the technology. *Biotechniques* **35**: 440–444.
32. Ferguson PL, Smith RD. 2003. Proteome analysis by mass spectrometry. *Annu Rev Biophys Biomol Struct* **32**: 399–424.
33. Simpson RJ, Hammacher A, Smith DK, Matthews JM, Ward LD. 1997. Interleukin-6: structure–function relationships. *Protein Sci* **6**: 929–955.
34. Balk SP, Ko YJ, Bubley GJ. 2003. Biology of prostate-specific antigen. *J Clin Oncol* **21**: 383–391.
35. Van Snick J, Cayphas S, Vink A, *et al*. 1986. Purification and $NH_2$-terminal amino acid sequence of a T-cell-derived lymphokine with growth factor activity for B-cell hybridomas. *Proc Natl Acad Sci USA* **83**: 9679–9683.
36. Van Snick J, Vink A, Cayphas S, Uyttenhove C. 1987. Interleukin-HP1, a T cell-derived hybridoma growth factor that supports the *in vitro* growth of murine plasmacytomas. *J Exp Med* **165**: 641–649.
37. O'Farrell PH. 1975. High resolution two-dimensional electrophoresis of proteins. *J Biol Chem* **250**: 4007–4021.
38. Klose J. 1975. Protein mapping by combined isoelectric focusing and electrophoresis of mouse tissues. A novel approach to testing for induced point mutations in mammals. *Humangenetik* **26**: 231–243.
39. Gorg A, Obermaier C, Boguth G, *et al*. 2000. The current state of two-dimensional electrophoresis with immobilized pH gradients. *Electrophoresis* **21**: 1037–1053.
40. Lottspeich F. 1999. Proteome analysis: a pathway to the functional analysis of proteins. *Angewandte Chem Int Ed* **38**: 2477–2492.
41. Jung E, Heller M, Sanchez JC, Hochstrasser DF. 2000. Proteomics meets cell biology: the establishment of subcellular proteomes. *Electrophoresis* **21**: 3369–3377.
42. Brunet S, Thibault P, Gagnon E, *et al*. 2003. Organelle proteomics: looking at less to see more. *Trends Cell Biol* **13**: 629–638.
43. Ramsby ML, Makowski GS, Khairallah EA. 1994. Differential detergent fractionation of isolated hepatocytes: biochemical, immunochemical and two-dimensional gel electrophoresis characterization of cytoskeletal and noncytoskeletal compartments. *Electrophoresis* **15**: 265–277.
44. Ramsby ML, Makowski GS. 1999. Differential detergent fractionation of eukaryotic cells. Analysis by two-dimensional gel electrophoresis. *Methods Mol Biol* **112**: 53–66.
45. Ji H, Baldwin GS, Burgess AW, *et al*. 1993. Epidermal growth factor induces serine phosphorylation of stathmin in a human colon carcinoma cell line (LIM 1215). *J Biol Chem* **268**: 13 396–13 405.
46. Travis J, Pannell R. 1973. Selective removal of albumin from plasma by affinity chromatography. *Clin Chim Acta* **49**: 49–52.
47. Pieper R, Su Q, Gatlin CL, *et al*. 2003. Multi-component immunoaffinity subtraction chromatography: an innovative step towards a comprehensive survey of the human plasma proteome. *Proteomics* **3**: 422–432.

Copyright © 2005 John Wiley & Sons, Ltd. 

48. Bjorck L, Kronvall G. 1981. Analysis of bacterial cell wall proteins and human serum proteins bound to bacterial cell surfaces. *Acta Pathol Microbiol Scand B* **89**: 1–6.
49. Bjorck L, Kronvall G. 1984. Purification and some properties of streptococcal protein G, a novel IgG-binding reagent. *J Immunol* **133**: 969–974.
50. Akerstrom B, Brodin T, Reis K, Bjorck L. 1985. Protein G: a powerful tool for binding and detection of monoclonal and polyclonal antibodies. *J Immunol* **135**: 2589–2592.
51. Guss B, Eliasson M, Olsson A, *et al*. 1986. Structure of the IgG-binding regions of streptococcal protein G. *EMBO J* **5**: 1567–1575.
52. Govorukhina NI, Keizer-Gunnink A, van der Zee AGJ, *et al.* 2003. Sample preparation of human serum for the analysis of tumor markers — comparison of different approaches for albumin and $\gamma$-globulin depletion. *J Chromatogr A* **1009**: 171–178.
53. Gorg A, Boguth G, Obermaier C, Weiss W. 1998. Two-dimensional electrophoresis of proteins in an immobilized pH 4–12 gradient. *Electrophoresis* **19**: 1516–1519.
54. Washburn MP, Wolters D, Yates JR III. 2001. Large-scale analysis of the yeast proteome by multidimensional protein identification technology. *Nat Biotechnol* **19**: 242–247.
55. Wu CC, MacCoss MJ. 2002. Shotgun proteomics: tools for the analysis of complex biological systems. *Curr Opin Mol Ther* **4**: 242–250.
56. Righetti PG, Castagna A, Antonioli P, Boschetti E. 2005. Prefractionation techniques in proteome analysis: the mining tools of the third millennium. *Electrophoresis* **26**: 297–319.
57. Zugaro LM, Reid GE, Ji H, *et al*. Characterization of rat brain stathmin isoforms by two-dimensional gel electrophoresis–matrix-assisted laser desorption/ionization and electrospray ionization-ion trap mass spectrometry. *Electrophoresis* **19**: 867–876.
58. Fountoulakis M, Juranville JF. 2003. Enrichment of low-abundance brain proteins by preparative electrophoresis. *Anal Biochem* **313**: 267–282.
59. Bjellqvist B, Ek K, Righetti PG, *et al*. 1982. Isoelectric focusing in immobilized pH gradients: principle, methodology and some applications. *J Biochem Biophys Methods* **6**: 317–339.
60. Egen NB, Bliss M, Mayersohn M, *et al*. 1988. Isolation of monoclonal antibodies to phencyclidine from ascites fluid by preparative isoelectric focusing in the Rotofor. *Anal Biochem* **172**: 488–494.
61. Bier M, Long T. 1992. Recycling isoelectric focusing: use of simple buffers. *J Chromatogr* **604**: 73–83.
62. Faupel M, Barzaghi B, Gelfi C, Righetti PG. 1987. Isoelectric protein purification by orthogonally coupled hydraulic and electric transports in a segmented immobilized pH gradient. *J Biochem Biophys Methods* **15**: 147–161.
63. Zuo X, Speicher DW. 2002. Comprehensive analysis of complex proteomes using microscale solution isoelectrofocusing prior to narrow pH range two-dimensional electrophoresis. *Proteomics* **2**: 58–68.
64. Righetti PG, Castagna A, Herbert B, Reymond F, Rossier JS. 2003. Prefractionation techniques in proteome analysis. *Proteomics* **3**: 1397–1407.
65. Hannig K. 1961. Die Tragerfreie Kontinuierliche Elektrophorese und Ihre Anwendung. *Fresenius Zeitschr Anal Chem* **181**: 244–274.
66. Hannig K, Heidrich HG. 1990. *Free-flow Electrophoresis*. GIT Verlag GmbH: Darmstadt, Germany.
67. Burggraf D, Weber G, Lottspeich F. 1995. Free flow-isoelectric focusing of human cellular lysates as sample preparation for protein analysis. *Electrophoresis* **16**: 1010–1015.
68. Zischka H, Weber G, Weber PJ, *et al*. 2003. Improved proteome analysis of *Saccharomyces cerevisiae* mitochondria by free-flow electrophoresis. *Proteomics* **3**: 906–916.
69. Hoffmann P, Ji H, Moritz RL, *et al*. 2001. Continuous free-flow electrophoresis separation of cytosolic proteins from the human colon carcinoma cell line LIM 1215: a non two-dimensional gel electrophoresis-based proteome analysis strategy. *Proteomics* **1**: 807–818.
70. Moritz RL, Ji H, Schutz F, *et al*. 2004. A proteome strategy for fractionating proteins and peptides using continuous free-flow electrophoresis coupled off-line to reversed-phase high-performance liquid chromatography. *Anal Chem* **76**: 4811–4824.
71. Bushey MM, Jorgenson JW. 1990. Automated instrumentation for comprehensive two-dimensional high-performance liquid chromatography of proteins. *Anal Chem* **62**: 161–167.
72. Wagner K, Racaityte K, Unger KK, *et al*. 2000. Protein mapping by two-dimensional high performance liquid chromatography. *J Chromatogr A* **893**: 293–305.
73. Henzel WJ, Billeci TM, Stults JT, *et al*. 1993. Identifying proteins from two-dimensional gels by molecular mass searching of peptide fragments in protein sequence databases. *Proc Natl Acad Sci USA* **90**: 5011–5015.
74. Pappin DJC, Hojrup P, Bleasby AJ. 1993. Rapid identification of proteins by peptide-mass fingerprinting. *Curr Biol* **3**: 327–332.
75. Henzel WJ, Watanabe C, Stults JT. 2003. Protein identification: the origins of peptide mass fingerprinting. *J Am Soc Mass Spectrom* **14**: 931–942.
76. Cargile BJ, Stephenson JL. 2003. An alternative to tandem mass spectrometry: isoelectric point and accurate mass for the identification of peptides. *Anal Chem* **76**: 267–275.
77. Baldwin MA. 2004. Protein identification by mass spectrometry: issues to be considered. *Mol Cell Proteom* **3**: 1–9.
78. Liotta LA, Ferrari M, Petricoin E. 2003. Clinical proteomics: written in blood. *Nature* **425**: 905.
79. Celis JE, Ostergaard M, Rasmussen HH, *et al*. 1999. A comprehensive protein resource for the study of bladder cancer: **http://biobase.dk/cgi-bin/celis**. *Electrophoresis* **20**: 300–309.
80. Celis JE, Wolf H, Ostergaard M. 2000. Bladder squamous cell carcinoma biomarkers derived from proteomics. *Electrophoresis* **21**: 2115–2121.
81. Nam MJ, Kee MK, Kuick R, Hanash SM. 2004. Identification of defensin $\alpha 6$ as a potential biomarker in colon adenocarcinoma. *J Biol Chem* **280**: 8260–8265.
82. Albrethsen J, Bogebo R, Gammeltoft S, *et al*. 2005. Upregulated expression of human neutrophil peptides 1, 2 and 3 (HNP 1–3) in colon cancer serum and tumours: a biomarker study. *BMC Cancer* (in press).
83. Ransohoff DF. 2005. Lessons from controversy: ovarian cancer screening and serum proteomics. *J Natl Cancer Inst* **97**: 315–319.

Copyright © 2005 John Wiley & Sons, Ltd. 
